# Changing patterns in chemical terrorism 1970–2021

**DOI:** 10.3389/fpubh.2025.1689809

**Published:** 2025-11-07

**Authors:** Rafael Castro-Delgado, Francisco Campillo Palma, Lucía Fernández-Arce, Helal Uddin, Ana Fernández-Somoano

**Affiliations:** 1 Department of Medicine, University of Oviedo, Oviedo, Spain; 2 Health Service Principality of Asturias (SAMU-Asturias), Research Group on Prehospital Care and Disasters (GIAPREDE), Health Research Institute of the Principality of Asturias (ISPA), Oviedo, Spain; 3 The University Institute of Oncology of Asturias (IUOPA), University of Oviedo, Oviedo, Spain; 4 Department of Sociology, East West University, Dhaka, Bangladesh; 5 CIBER de Epidemiología y Salud Pública (CIBERESP), Instituto de Salud Carlos III (ISCIII), Madrid, Spain

**Keywords:** chemical hazard release, terrorism, mass casualty incidents, chemical terrorism, public health preparedness, incident trends

## Abstract

**Introduction:**

Chemical terrorism remains a major public health threat due to its acute and chronic effects, requiring coordinated response efforts. Although decontamination methods and training have improved, uncertainties persist, particularly as chemical agents evolve. This study examines incidents of chemical terrorism worldwide from 1970 to 2021, analyzing changes in pattern trends and typologies to earlier data, aiming to measure shifts in frequency, geographic distribution, and toxic chemical usage.

**Methods:**

Data from the Global Terrorism Database (GTD) was used. Absolute frequencies and percentages were calculated to describe the distribution of the variables over time. Temporal trends were assessed using simple linear regression, reporting the coefficient of determination (r^2^) and *p*-values. Variables analyzed included incident frequency (yearly and monthly), geographic region, duration, single versus multiple attacks, success rates, suicidal intent, organizational claims, and toxin types. The number of deaths, injuries, and property damage was also analyzed. Data from 1970 to 2015 and 2016–2021 were compared to identify shifts in patterns.

**Results:**

A total of 353 chemical terrorism cases were identified. Between 2016 and 2021, incidents declined, reversing the upward trend observed from 1970 to 2015. The most affected areas were South Asia and Middle East/North Africa. Most attacks lasted under 24 hours, involved a single incident, and had a 77.84% success rate. Nerve agents and organophosphates were among the most used (13.35%) toxins and caused the highest mortality (18.18%) and injury rates (63.33%).

**Conclusion:**

Chemical terrorism incidents have decreased in recent years. However, these incidents still pose risks to human life and property. Training for first responders should prioritize the detection and management of nerve agents and organophosphates. Improved detection systems and standardized protocols are imperative for strengthening response effectiveness in future incidents.

## 1 Introduction

The threat of chemical terrorism remains a major global security concern. Different authors have highlighted the necessity for better response protocols to address chemical hazards during terrorist incidents (1). Despite improvements in decontamination methods and responder training, uncertainties remain due to the changing nature of chemical agents (2). Mass casualty incidents (MCIs) including chemicals show unique challenges, as such events can overwhelm healthcare systems, resulting in human and material damage (3). To address these threats, a thorough understanding of the types, patterns, and impacts of chemical terrorism is required.

Since 1970, the Global Terrorism Database (GTD) has documented chemical terrorist incidents globally (4). GTD define terrorism as “*the systematic threat or use of violence, by non-state actors, whether for or in opposition to established authority, with the intention of communicating a political, religious or ideological message to a group larger than the victim group, by generating fear and so altering (or attempting to alter) the behavior of the larger group*” (4). Although data is available, there have been limited studies focusing on the analysis of trends over the past decade. The database contains comprehensive information on terrorist incidents, covering variables such as attack success rates, geographic distribution, and toxic chemical types. Previous studies have shown the importance of this data in improving response preparedness, but much of the research is either outdated or lacks depth in evaluating recent incidents (2).

Chemical incidents, whether accidental or intentional, have far-reaching consequences. Globally, thousands of people are affected by chemical spills, fires, and terrorist attacks including hazardous materials each year (2). Intentional chemical releases, particularly for terrorist purposes, have increased, placing general populations at risk. The 2018 Novichok attack in Salisbury is a notable example of how chemical agents can bring wide-scale harm, with long-lasting impacts on both individuals and communities (2). Moreover, attacks on industrial chemical facilities pose an added layer of threat, as these incidents can result in the release of hazardous substances on a massive scale (2).

Although the use of water for decontamination remains common in chemical incidents, recent studies challenge its efficiency. Evidence-based study suggests that water-based decontamination can increase the absorption of lipophilic chemicals, potentially increasing harm (5). Recent protocols emphasize the need for alternative methods, such as dry decontamination, which has been found effective for certain toxic agents (6). Despite these improvements, no standardized global approach exists, and practices vary between countries (2). Previous studies have argued that timely removal of contaminated clothing and rapid decontamination is crucial for improving survival rates (7). However, there are still research gaps in areas such as the treatment of chemically contaminated patients and the public’s perception of different decontamination methods (2).

Some security organizations have acclaimed the changing chemical threats, from traditional agents like sarin to emerging toxic chemicals such as Novichok and pharmaceutical-based agents (8). These developments make difficult toxic detection and response, underscoring the importance of continuous improvement in decontamination strategies. Training first responders to recognize and manage these agents remains vital (9). Also, surveillance programs may be essential for early detection, and artificial intelligence and machine learning may have a crucial role to make improvement in this sense (10, 11). Yet, there is still limited evidence on the validity of existing triage systems for chemical MCIs (3).

The public health implications of chemical terrorism are far-reaching, as these incidents not only cause immediate casualties but also result in long-term health consequences for affected populations (12). Exposure to toxic agents can lead to acute poisoning, chronic illnesses, and psychological trauma, placing a significant burden on healthcare systems (12). Additionally, chemical attacks can contaminate air, water, and food supplies, necessitating a coordinated public health response to mitigate secondary exposure and environmental damage. Given these challenges, enhancing preparedness, response, and recovery strategies is critical to minimizing health risks and strengthening community resilience against chemical (12) or any other threats (climate change, natural disasters, etc.). A comprehensive analysis of chemical terrorist incidents is required to design the formulation of future policy and measures to enhance preparedness, thereby facilitating improvements in public health awareness and response with respect to incidents related to chemical terrorism.

This study, by examining the GTD data over a 50-year period (1970–2021) and focusing on the period from 2016 to 2021, aims to identify changes in incident patterns and offer insights into the current state of chemical terrorism, eventually contributing to more effective response strategies.

## 2 Methods

### 2.1 Study design

This study applied a retrospective, observational approach to examine trends in chemical terrorism incidents over a 50-year period (1970–2021) using data from the GTD. The analysis identified trends in the frequency, geographic distribution, and types of chemical agents used in terrorist attacks.

### 2.2 Data collection

The study retrieved data from the GTD, applying filters to identify chemical weapon-related terrorist incidents. No filters were applied in terms of region, country, or perpetrator group in order to include all events worldwide. All incidents involving chemical weapons were included, excluding non-state actors’ incidents as they are not listed in GTD. Failed and/or undetected terrorist incidents are not included.

Prior to analysis, duplicate entries in the GTD dataset were removed. No imputation or additional treatment was applied to missing data; missing values were left as provided in the original dataset.

Each incident was grouped based on variables such as year, month, country, attack type, target type, and type of chemical agent used. Incidents were also coded for specific variables, including whether the attack was part of a multiple-event sequence, whether it involved suicide tactics, and whether it was successful. Variables such as deaths, injuries, and property damage were tracked to measure the impact of each attack.

### 2.3 Data coding and definitions

Variables from the GTD were recorded for consistency with the study’s objectives. Key variables included:

Year/Month of Incident: Numeric variables capturing the date of the incident.
Geographic Location: Categorical variables that identify the country and region with the aim of organizing information from all locations around the world.
Duration: Binary variable indicating whether the attack lasted more than 24 hours (h).
Type of Chemical Agent: A new categorical variable was created to classify chemical agents into categories such as cyanide, tear gas, incendiary, poison, nerve agents, mercury, and chlorine gas.
Outcome: Variables recording deaths, injuries, and property damage (resulted or not resulted in property damage).

Incidents are included in GTD only if they met two of the three criteria for terrorism: (1) aiming to achieve a political, economic, religious, or social goal; (2) intending to coerce or intimidate; and (3) falling outside legitimate warfare.

### 2.4 Data analysis

Descriptive analysis was conducted across three timeframes: 1970–2021, 1970–2015, and 2016–2021. Trends in the frequency of chemical attacks, the geographic distribution of incidents, and the types of chemical agents used were examined. Specific consideration was paid to the success rate of attacks, the involvement of suicide tactics, and the severity of outcomes (deaths, injuries, and property damage).

Additionally, the study compared the prevalence and impact of specific types of chemical agents, focusing on the most frequently used toxic chemicals, such as nerve agents and organophosphates, which were associated with the highest mortality and injury rates. A *p*-value less than 0.05 (*p* < 0.05) was considered statistically significant. All statistical analyses were performed using Microsoft Excel and Stata version 14 (StataCorp, College Station, TX, USA).

### 2.5 Statistical approach

The primary analysis was descriptive, focusing on the distribution of incidents across time and geographical locations. Trend was analized using linear regression. Numbers and percentages for chemical agent types and their outcomes (e.g., deaths, injuries, and property damage) were obtained. It was also examined how the use of specific chemical agents changed over time and across different geographic regions.

## 3 Results

### 3.1 Incidents over time

**Figure 1:**
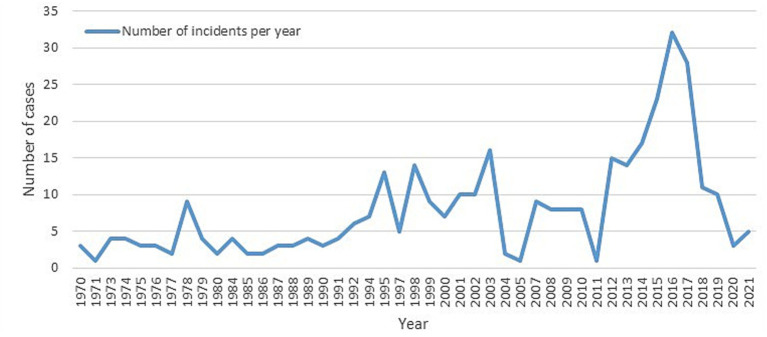
Incidents over time.

**Figure 2:**
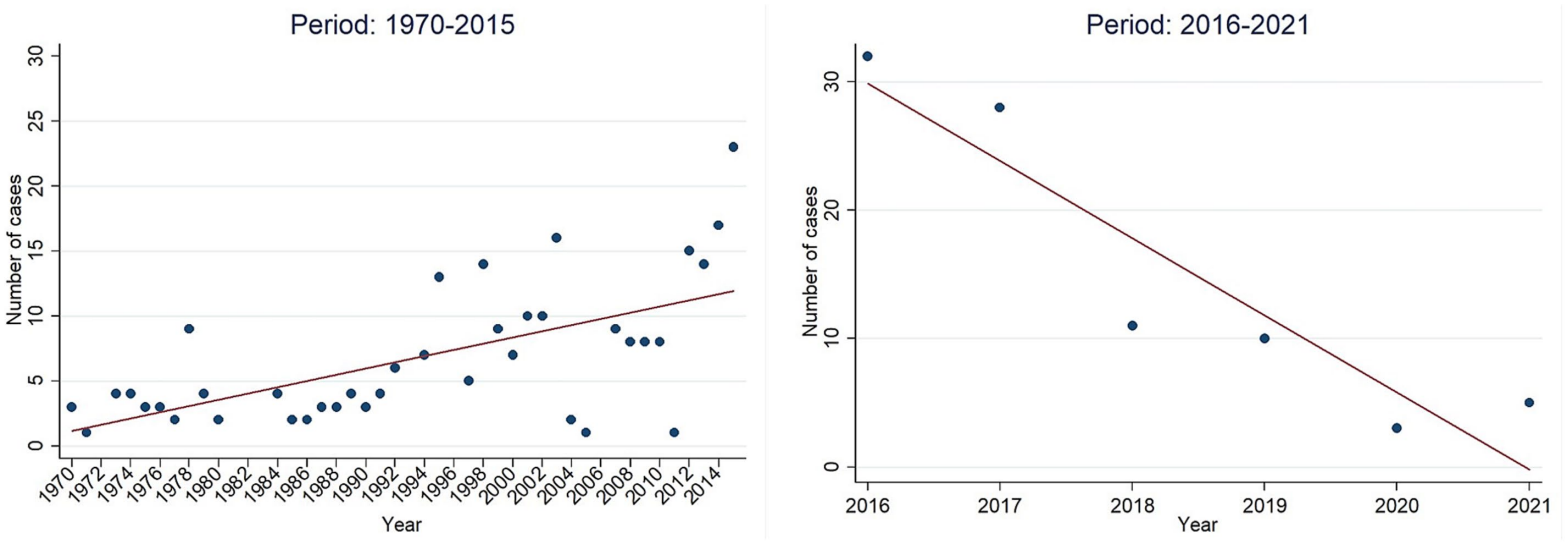
Incidents trend. Period 1970–2015 vs. 2016–2021.

A total of 353 cases were identified after removing duplicate entries, with a total of 44 deaths and 996 injured (7 and 500, respectively, in Sarin attack in Tokyo). Between 1970 and 2011, the number of chemical terrorist incidents fluctuated, with four notable peaks in 1978, 1995, 1998, and 2003 (Figure 1). A notable rise in incidents occurred from 2011 to 2016, topping at 32 incidents in 2016. However, from 2016 to 2021, there was an observed decline, reaching just 3–5 incidents per year by 2020–2021. The scatter plots show this change clearly, with a statistically significant direct linear relationship for 1970–2015 (r^2^ = 0.387, *p* < 0.001) and a strong negative trend for 2016–2021 (r^2^ = 0.856, *p* = 0.008) (Figure 2), indicating a reduction 27 incidents since 2016 (average per year = 5.4; standard deviation = 7.3; 95% confidence interval [−2, 13].

**Figure 3:**
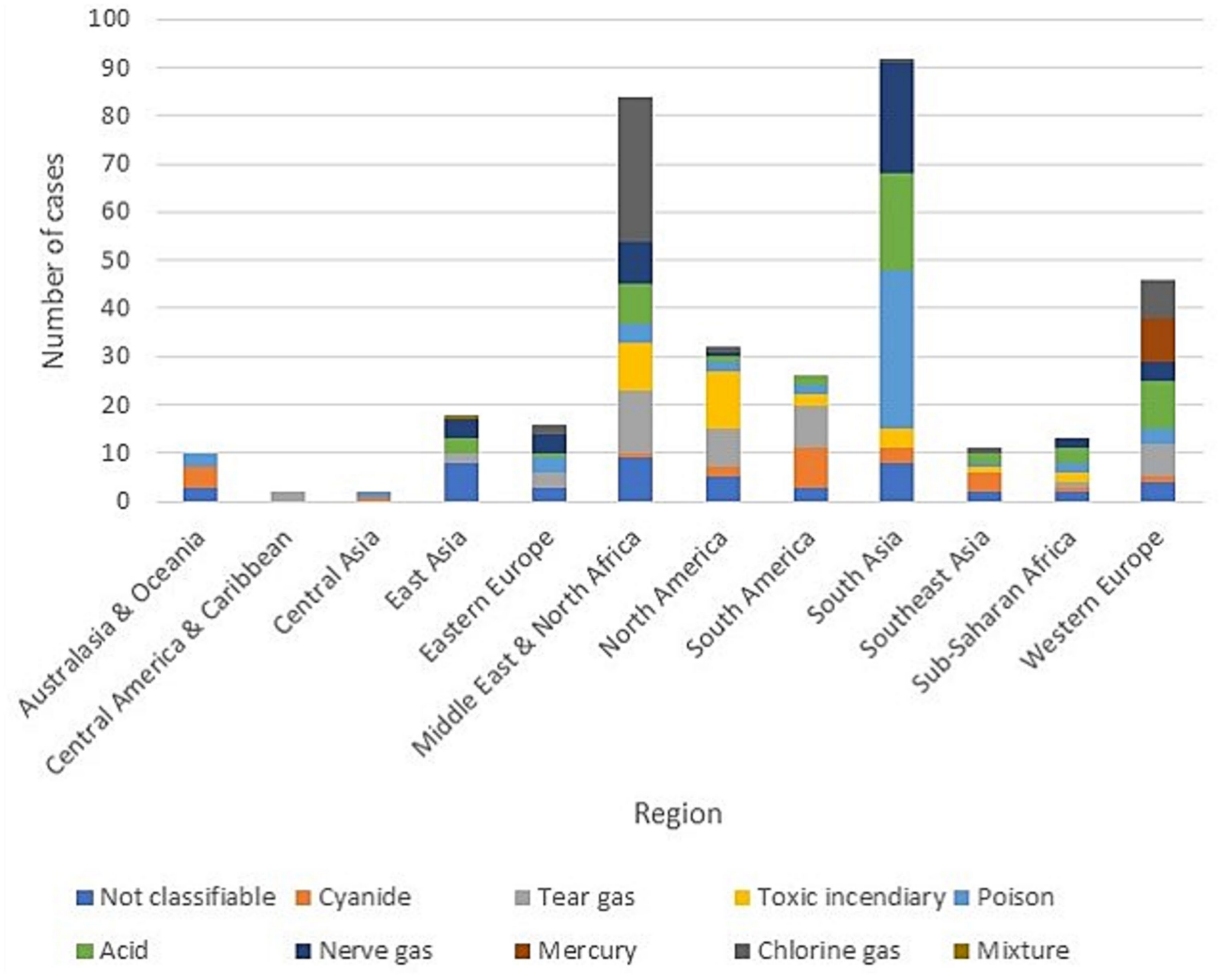
Type of chemical agent by geographical distribution.

South Asia and the Middle East/North Africa accounted for the highest number of incidents (50% of the total from 1970 to 2021). Central America/Caribbean and Central Asia experienced the fewest incidents, contributing only 1.1% (Figure 3). Chemical terrorist incidents were most frequent between March and June, with around 40 incidents during these months, compared to about 20 incidents in other months. November served as an intermediate month with 29 incidents.

**Figure 4:**
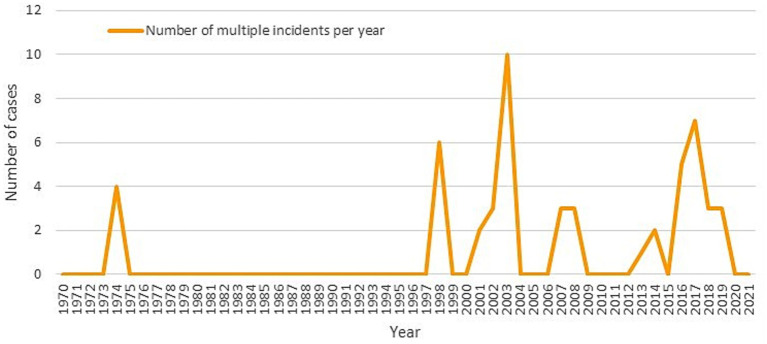
Multiple attacks over time.

Most incidents (98.86%) were solved within 24 h, with only 1.14% lasting longer. These three incidents in the years 2013, 2018 and 2019 were kidnapping in which poison food or drugs were used in the attacks, two of them against police Security Forces/Officers in Iran and Afghanistan. Most incidents (85.23%) involved a single attack, while 14.77% included multiple attacks within the same incident. Figure 4 highlights the appearance of multiple incidents in the late 1990s.

**Figure 5:**
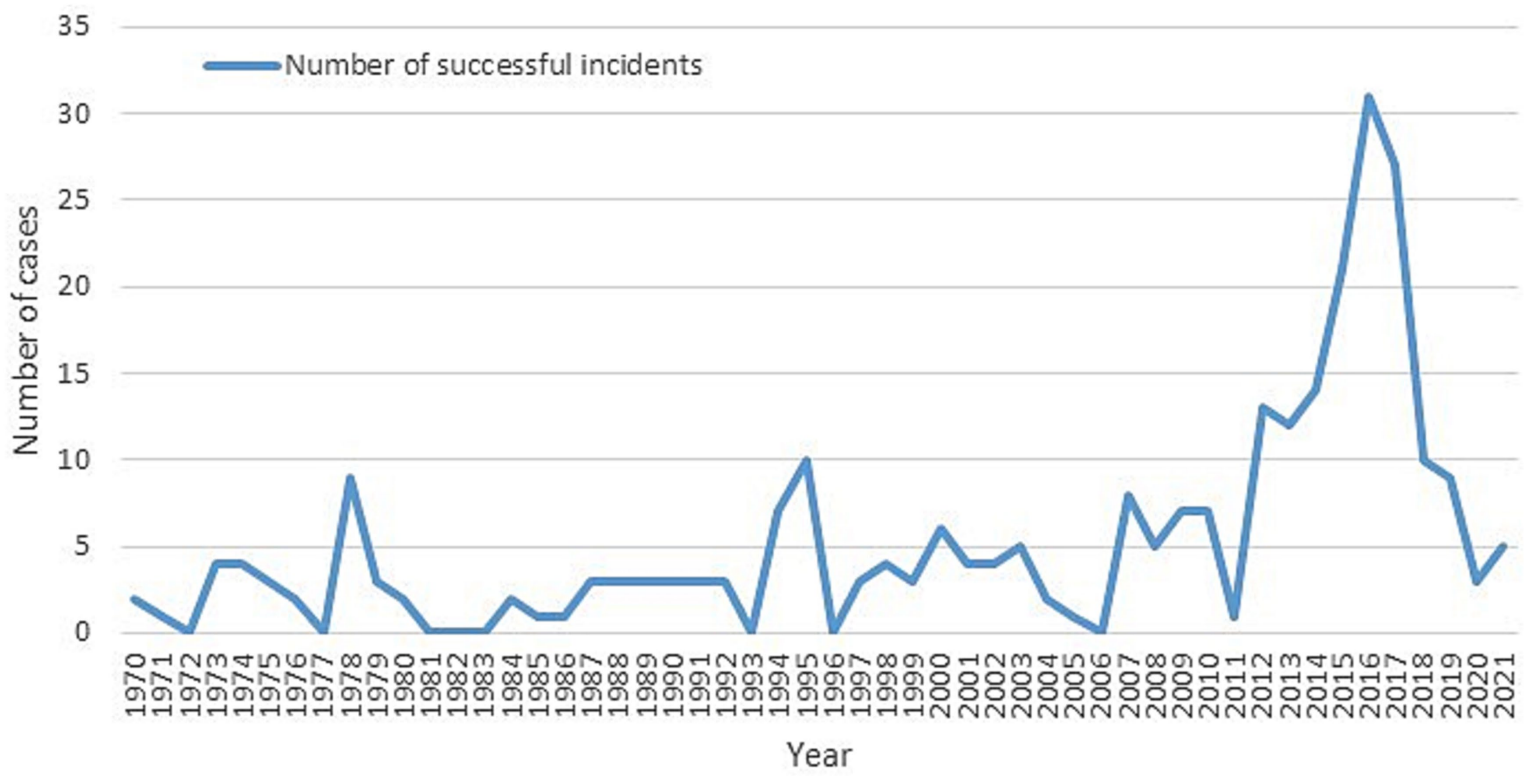
Success rate.

Around 77.84% of chemical terrorist incidents were deemed successful, causing damage, with a peak in successful incidents around 2016–2017. The number of successful incidents declined in subsequent years (Figure 5). Only 1.99% of the incidents involved suicide bombers, and just 14.07% of attacks were claimed by organizations. Related to the way of using these toxic chemicals, 97 (27.63%) used them as a poison, and 73 (20.79%) using explosive devices to spread them. In this context, the term ‘poisons’ refers to the classification used in the database (e.g., cyanide, arsenic, strychnine), whereas acids and nerve agents/organophosphates were reported as separate categories.

**Figure 6:**
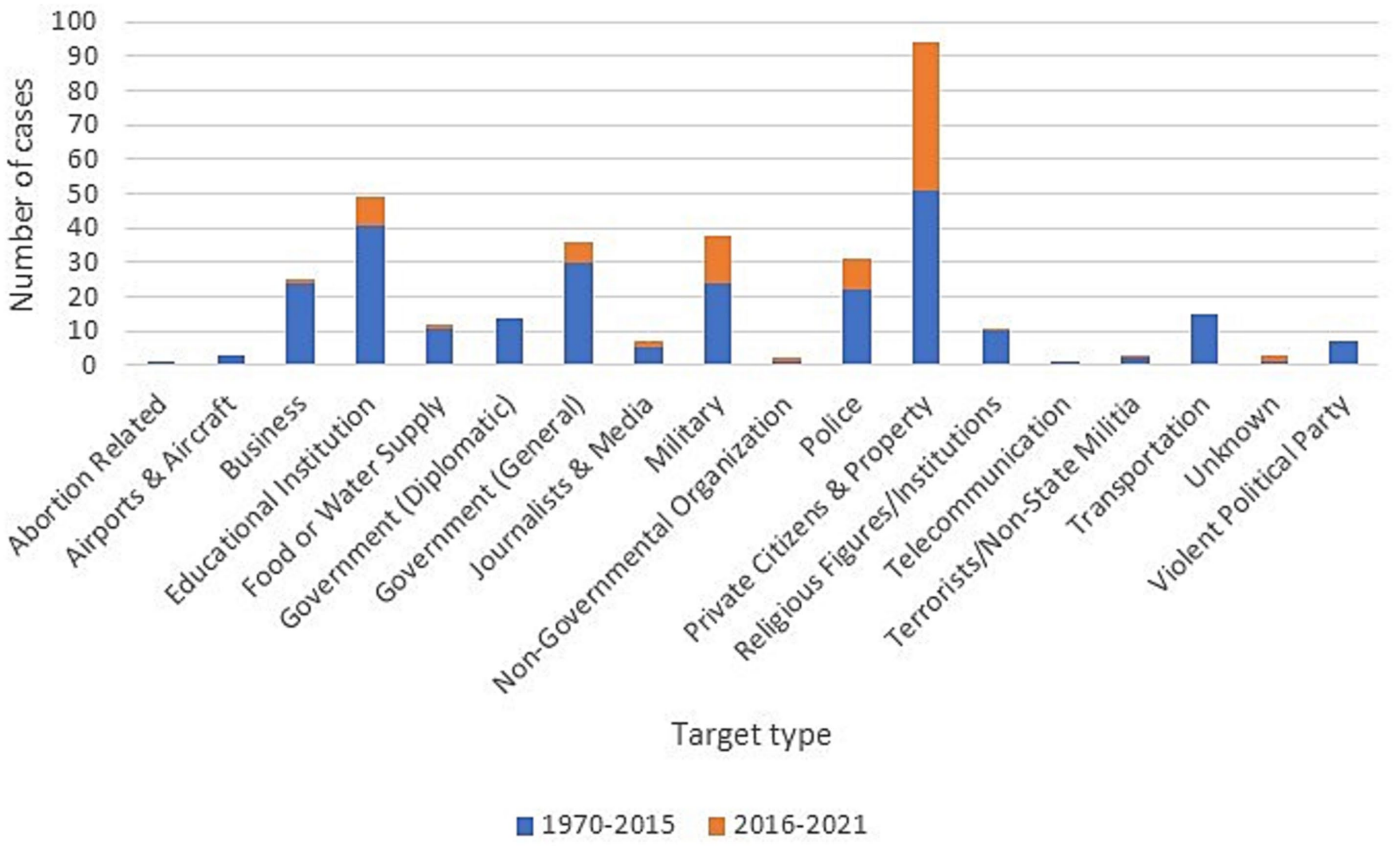
Number of incidents by target type and period.

Private citizens and structures were the primary targets, making up 26.70% of incidents. Educational institutions (13.92%), government structures (10.23%), and military targets (10.80%) followed. Other targets, such as religious institutions and transport systems, each accounted for less than 10% (Figure 6).

**Figure 7:**
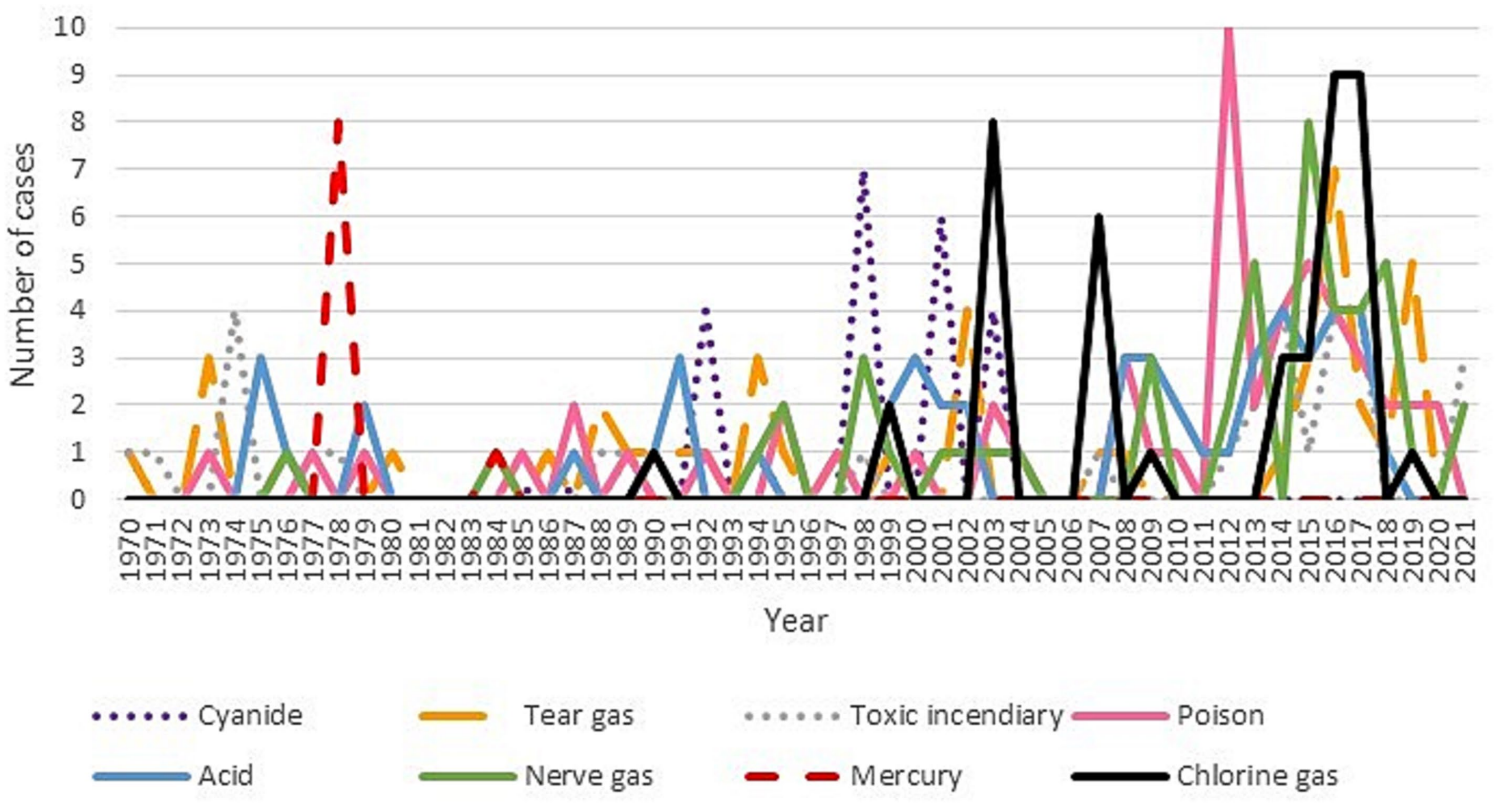
Chemical agents used over time.

Poisons were the most common toxic chemicals (15.34%), followed by acids (14.20%) and nerve agents and organophosphates (13.35%). Mercury and cyanide were used less frequently, accounting for 2.56 and 7.10%, respectively. Figure 7 shows the temporal trends for specific chemicals, with nerve agents peaking in 2015 and chlorine gas incidents rising in 2016–2017.

Incidents involving unknown toxic chemicals had the highest fatality rate (47.06%). Nerve agents and organophosphates also had weighty mortality (18.18%). Injuries were most common in incidents involving nerve agents, with 68.33% of cases causing harm. Property damage was most associated with mercury (66.67%) and incendiary toxic chemicals (60.00%) (Table 1).

**Table 1:** Average number of deaths and injuries by chemical agent.

TipoTox	Deaths			Injuries		
	Mean	Mode	Max	Mean	Mode	Max
Not classifiable	0.47	0	200	0.11	0	671
Cyanide	0.05	0	15	0.01	0	53
Tear gas	0.13	0	65	0.15	0	1,500
Toxic incendiary	0.08	0	25	0.02	0	130
Poison	0.17	0	32	0.19	0	301
Acid	0.07	0	25	0.02	0	46
Nerve gas	0.18	0	60	0.68	0	5,500
Mercury	0.00	0	0	0.00	0	5
Chlorine gas	0.13	0	36	0.06	0	250
Mixture	0.00	0	1	0.00	0	1

## 4 Discussion

The GTD indicated a consistent upward trend in chemical terrorist incidents until 2016. Since then, the frequency has gradually decreased. The exponential increases observed up until 2016 were noted previously (7, 13).

It is now crucial to examine the decline between 2016 and 2021 to determine whether this reduction reflects a genuine decrease in terrorist activity or if other factors may be at play. It is essential to continue surveillance to monitor this trend. Some causes may be related to an improvement in intelligence mitigation activities and early detection of threats by police and security forces, but also with the international pressure against terrorist groups.

While the total number of incidents has dropped, the success rate of attacks remains high, with over 90% of chemical terrorist incidents successfully causing damage. This may suggest that terrorist organizations are more efficient, requiring fewer attempts to achieve high levels of destruction. More reasons behind this decline remain unclear but could be linked to development policies, lessons from past events, or other unstudied factors (14). Future studies may help find the causes of this trend. Nonetheless, the need for improved healthcare and emergency response systems remains, especially given the persisting gaps in training and preparedness for such incidents (9, 15). It is also important to note that COVID pandemic may have had some influence as there is a slight decrease from 2019 to 2020 that maintained in 2021, but as decreased trend start in 2016, most probably COVID has not affected this global decreased trend (16).

Despite the decreased frequency of chemical terrorist incidents, their potential impact is something to consider. Most incidents are resolved within 24 h, yet three-quarters of these events result in some form of damage.

The trend of single attacks still dominates (85%), but multiple attack incidents have increased since the 1990s, potentially heightening their impact on emergency systems. These attacks also cause damage to public health, which has been less extensively studied (13). Ezcayez et al. (17) organize their research into three levels: individual, community health and environmental effects. Studies indicate that some attacks continue to have repercussions 30 years later or have led to deterioration in health services (16). Future studies could explore how incidents are timed around relevant events or vulnerable periods, such as religious holidays or political elections, to improve incident prevention strategies.

Targeting general population remain the primary focus of chemical terrorist attacks, with the highest frequency seen between 2014 and 2020. In their review up to 2015, Santos et al. (18) found that 19% of the targeted population was private, but from 2016 onwards, as noted in the study by DeLuca et al (13), this percentage increased to 26% trend that was also observed in our study up to 2021. Educational institutions also appeared to be frequent targets, likely due to the vulnerability of children and the potential for widespread disruption of a nation’s educational infrastructure although their proportion has decreased in favor of the aforementioned targets. Understanding the rationale behind targeting civilians and education could help develop preventative measures.

Geographically, the majority of chemical terrorist incidents occurred in South Asia and the Middle East/North Africa, which together account for 50% of the total incidents from 1970 to 2021. Particularly in the Middle East/North Africa, 49% of these incidents occurred in just 2 years, 2016 and 2017. This concentration warrants further investigation into the sociopolitical conditions that contributed to this spike. Zakaria et al. (19) conducted a study in the Middle East/North Africa region and concluded that country-specific vulnerabilities are evident when chemical weapons are used as a tool of terror in long-term conflicts. The study highlights that, beyond immediate harm, long-term problems such as health system strain, social disruption, and conflicts with the government may arise (9).

The regional distribution of specific toxic chemicals also varied, with organophosphates and nerve gases appearing frequently across most geographic areas. These agents are known to cause serious injuries, as 68% of incidents involving organophosphates resulted in injuries. This aligns with the literature on the harmful nature of these chemicals, further emphasizing the need for improved management and detection capabilities among first responders.

When examining the toxic chemicals used in these incidents, poisons, acids, nerve gases, and organophosphates were the most frequently used chemicals, comprising approximately 68% of the total. No single toxic chemical dominates, suggesting that emergency systems should be prepared to handle a wide range of chemicals. Mercury and cyanide were used less frequently, but the potential for unknown or newly modified chemicals remains a concern. The third most common category involves unknown toxic chemicals, which may represent either unclassified chemicals or new agents specifically designed to evade detection. This highlights the need for better detection technologies and first responder training. These unknown toxics would deserve a more in deep surveillance in future research as they may represent new emerging agents to bear in mind.

Mortality and injury rates were notably higher in incidents involving unknown toxic chemicals, underscoring the importance of timely identification of the chemical involved. For example, Tokio Sarin attack ranked the second with regards to injury rates (16). Delays in detection can prevent the effective application of specific decontamination methods or antidotes, leading to higher death rates. Enhancing detection methods could have a profound impact on reducing mortality in future incidents.

In terms of property damage, mercury, while rarely used, caused the most structural damage, whereas organophosphates, which rank high for mortality and injuries, resulted in minimal property damage. This inverse relationship suggests that response strategies should be tailored not only to reduce human casualties but also to mitigate infrastructure damage, depending on the chemical involved.

The study of these new agents must be continuous, as demonstrated by the 2018 report issued by the Organization for the Prohibition on Chemical Weapons (OPCW) that mention the potential use of fentanyl and its non-pharmaceutical analogues (20). When deployed in aerosol form, these substances could be illicitly used for terrorist or wartime purposes (21). Additionally, there is a need to enhance the management of products used in vulnerable populations, which includes the development of specific protocols and guidelines (22). New approaches using computing simulation are showing new challenges related to prehospital management, opening the discussion related to stay and play or scoop and run, as based on these findings quick transport plus on-site antidote administration may save most lives (23).

When comparing with conventional terrorism, some authors have recently found that global terrorism risk has evolved over the past five decades with four distinct stages: emerging stage (1970–1991), descending stage (1992–2000), rampant stage (2001–2014), and attenuating stage (2015–2020), with 117 countries showing an increasing trend, and 56 countries showing a decreasing trend in terrorism risk (24). Our findings in recent chemical terrorism (2016–2021) may be similar as their attenuating stage.

By analyzing trends and toxic characteristics, public health authorities can adapt specific procedures to specific threats (19). However, it is imperative to acknowledge that global health system preparedness is not the sole consideration relevant to this issue. Indeed, civilian preparedness must be given due consideration by the public health authority (25).

These findings emphasize the need for a cohesive, cross-sectoral strategy involving governments, medical institutions and global agencies, as well as local communities. In its 2023 report, the United Nations states that it continues to dedicate important efforts to working in high-incident areas with their local governments (14). This holistic approach must focus on immediate prevention and response mechanisms, while also investing in the recovery, rehabilitation and resilience of affected populations.

### 4.1 Strength and limitations

The strengths of this study include extensive analysis of data over a 50-year period from the GTD, which offers comprehensive information on chemical terrorism incidents. This longitudinal approach facilitates a meaningful comparison of trends in incident frequency, geographic distribution, and types of chemical agents, suggesting valuable insights into changing patterns. However, there are some limitations to consider. It is acknowledged that there may be potential inconsistencies in the data coding and gaps in the reporting, particularly in recent years. Additionally, the GTD data does not describe all nuances of regional variations or incident specifics, which may affect the generalizability of findings. Hence, further research is needed to address the gaps in our understanding of these incidents, particularly regarding the use of unknown toxic chemicals and the regional variations in chemical use. This is essential in the case of state-terrorism, not included in GTD and that may be an important source of unknown chemicals. Other limitation is the fact that failed and/or undetected terrorist incidents are de facto not included in the database, but these are worth considering when planning for a response against terrorist incidents as they could give information related to new types based on attempts. In this sense, the trend of failed terrorist incidents could be an indicator of improved prevention and detection, aspects that cannot be included in our research results and discussion. Other limitation is that we have not analyzed specific seasonal influence modified by regional context related to summer/winter north and south.

## 5 Conclusion

In sum, while the GTD captures a recent decline in the frequency of terrorist incidents including toxic chemicals in the period 2016–2021, the potential for important harm to human life and property persists. This study highlights specific characteristics of these incidents, including their geographical concentration, seasonal peaks, and resolution within 24 h, alongside their typical lack of suicidal intent or claims of responsibility. Additionally, the diverse objectives of these events complicate the understanding of their underlying motivations. Given the relatively low property damage but considerable risks to human health, it is imperative that governments improve detection systems and train first responders in identifying potential toxic exposures based on toxic syndromes, specific awareness information and on-scene surveillance approach, as they may the first ones to arrive to an unexpected chemical attack. Prioritizing the detection and management of nerve gases and organophosphates, the most common agents linked to fatalities and injuries, will notably improve response protocols and overall public safety. A public health approach will improve health system awareness and response based on chemical terrorism trends.

## Data Availability

The original contributions presented in the study are included in the article/supplementary material, further inquiries can be directed to the corresponding author.
